# The average gonadotrophin dosage per follicle is predictive of ovarian response and cumulative live birth chances after in vitro fertilization: a retrospective cohort study

**DOI:** 10.1186/s12905-023-02195-5

**Published:** 2023-02-04

**Authors:** Shan Liu, Shuai Ma, Yuan Li

**Affiliations:** 1 Medical Center for Human Reproduction, Beijing Chao-Yang Hospital, Capital Medical University, 8 Gongren Tiyuchang Nanlu, Chaoyang District, Beijing, China; 2 Present Address: Jinxin Fertility Group, No. 301, North Jingsha Road, Jinjiang District, Chengdu, Sichuan China

**Keywords:** In vitro fertilization, Ovarian stimulation, Ovarian response, Pregnancy outcome

## Abstract

**Background:**

With the development of assisted reproduction technology (ART), many indicators have been proposed to evaluate ovarian response, and then predict pregnancy outcomes. In general, the predictive values remain limited.

**Objective:**

To further explore the indicators to evaluate ovarian sensitivity to gonadotrophin (Gn) stimulation more accurately.

**Methods:**

This retrospective cohort study included 330 women who underwent an entire ART cycle. We aimed to assess whether a new index, termed as average Gn dosage per follicle, could be used as a marker for ovarian response and pregnancy outcomes. It was calculated as the ratio of total Gn dose during ovarian stimulation and the number of pre-ovulatory follicles (PFC) on the trigger day. Patients were divided into three subgroups according to the average Gn dosage per follicle: below the 33rd percentile (Group A), between 33rd and 67th percentiles (Group B), and above the 67th percentile (Group C). Then stimulation data, laboratory and clinical outcomes were compared among the groups.

**Results:**

The results showed patients in Group A had the best ovarian response, the number of retrieved oocytes was significantly higher than in Group B and C. A multivariate regression analysis showed that average Gn dosage per follicle was an independent predictor of cumulative live birth rates (CLBRs) [adjusted odds ratio (OR): 0.96, 95% confidence interval (CI): 0.95–0.98, *P* < 0.01].

**Conclusions:**

The present study showed that average Gn dosage per follicle appears to be a highly reliable index of ovarian response to exogenous Gn and can be useful to estimate CLBR.

**Supplementary Information:**

The online version contains supplementary material available at 10.1186/s12905-023-02195-5.

## Introduction

Ovarian stimulation is a crucial step in assisted reproduction technology (ART), which aims to retrieve a certain number of high-quality oocytes for subsequent in vitro fertilization (IVF) and allow selection of best quality embryos for transfer. Usually, the number of oocytes retrieved is the parameter most often used to evaluate the response of ovary to gonadotropins (Gn) stimulation. Previous studies have demonstrated that oocyte number is closely related to live birth rate (LBR) in ART [1]. While the aim of any ovarian stimulation is to get the optimal number of oocytes, it may not be achievable for all patients. Based on number of oocytes retrieved, patients are usually classified as high (hyper), normal or poor responders [2]. Besides, hypo-responders with impaired response to Gn also exist [3, 5]. Accurate classification relies primarily on ovarian reserve assessment prior to the initial of cycle. Commonly used indicators of ovarian reserve include age, basal hormone level including follicle-stimulating hormone (FSH), estradiol (E_2_), Anti-Mullerian Hormone (AMH), and antral follicle count (AFC).

With the development of ART, many indicators have been proposed in recent years, such as follicle output rate (FORT), follicle-to-oocyte index (FOI), ovarian sensitivity index (OSI), to evaluate ovarian response/sensitivity, and then predict pregnancy outcomes [4, 6–8]. Although all these indexes seem to be useful in the evaluation of ovarian response, some drawbacks should not be ignored. In general, the predictive values remain limited, and unexpected ovarian response often occur during stimulation. For example, some patients show a "slow response" to FSH, with slow estrogen growth and follicle development, or require a larger dose of Gn, which is inconsistent with age, BMI, ovarian reserve and other indicators [9–11]. In a population of women of advanced age with unexplained infertility, FOI, FORT and OSI do not show a stronger or more informative association with live birth than the components used for their calculation, i.e., the number of oocytes retrieved, the AFC, the number of pre-ovulatory follicles (PFC) and the FSH total dose [12].

Taken together, it is necessary to further explore the indicators reflecting ovarian response or to use multiple indicators in combination, to evaluate ovarian sensitivity to Gn stimulation more accurately. It has been observed that both total Gn dosage and PFC are important measures of ovarian response. We assume a ratio of them, termed as “average Gn dosage per follicle”, is a better representation of ovarian response rather than either parameter on its own. This retrospective study was carried out to assess the use of average Gn dosage per follicle as a marker of ovarian response during stimulation, and predict pregnancy outcomes.

## Methods

### Study design and participants

This was a single-center retrospective cohort study. A total of 330 infertile females underwent an entire ART cycle between June 2018 and June 2020 were enrolled. All the patients were entirely treated in Beijing Chao-Yang Hospital, Capital Medical University. The study was approved by the Ethics Committee of Beijing Chao-Yang Hospital, Capital Medical University (No. 2021-SCI-61). Written informed consent was waived by the Ethics Committee of Beijing Chao-Yang Hospital, Capital Medical University due to the retrospective nature of the study. Analyses of data was performed in accordance with the rules and regulation with approvals from the Ethics Committee of Beijing Chao-Yang Hospital.

The inclusion criteria were as following: (i) infertile duration over 1 year; (ii) 23 years ≤ female age ≤ 38 years; (iii) a spontaneous menstrual cycle length of 21–35 days; (iv) 18 kg/m^2^ ≤ body mass index (BMI) ≤ 28 kg/m^2^; (v) baseline FSH ≤ 10mIU/mL; (vi) treated with gonadotropin releasing hormone (GnRH) antagonist protocol. The exclusion criteria were: (i) polycystic ovary syndrome; (ii) endometrium diseases including endometrial atypical or complex hyperplasia, endometrial polyp and intrauterine adhesion; (iii) adenomyosis; (iv) history of ovarian or uterine surgery; (v) spontaneous abortion 3 times or more (including biochemical pregnancy loss).

Average Gn dosage per follicle was calculated as the ratio of total administered FSH dosage during ovarian stimulation and PFC. Patients were divided into three subgroups accordingly: below the 33rd percentile (Group A), between 33rd and 67th percentiles (Group B), and above the 67th percentile (Group C). There were 110 patients in each group. The use of 33rd, 67th percentile is chosen for statistical purpose and can be done automatically by statistical software.

### Ovarian stimulation, oocyte retrieval, and embryo transfer

All patients received flexible GnRH antagonist protocol. Ovarian stimulation was started by administering recombinant FSH (rFSH; Gonal F, Merck Serono, Germany) at a daily dose of 150–225 IU from menstrual cycle day 3 for 4–5 days. The initial dose of Gn was decided based on age, BMI, basal FSH and AFC. Ovarian response to stimulation was monitored by transvaginal ultrasound examination and hormone measurement from stimulation day 6. From day 6, Gn administration dose was adjusted according to ovarian response. GnRH antagonist (Cetrotide, Merck Serono, Germany) 0.25 mg daily was added when diameter of the maximal follicle was over 14 mm or the serum E_2_ reached 300 pg/ml. The co-treatment continued until the trigger day.

Triptorelin (Decapeptyl, Ferring, Paris, France) 0.2 mg and human chorionic gonadotropin (hCG, Lizhu Pharmaceutical, Guangzhou, China) 2000–3000 IU were injected for the final maturation when more than 2 follicles reached 18 mm in diameter. Ovum pick-up (OPU) was conducted 36 hours later and the oocytes were fertilized by either IVF or ICSI, depending on sperm quality.

Cleavage embryos on third day after fertilization (D3) were graded by morphological criteria on the basis of the number and size of blastomere and the percentage of fragmentation. Good quality embryos were defined as those reach 7–9 cells, with < 20% fragmentation and no multinucleation. Up to two good quality cleavage fresh embryos were transferred to the uterus on D3. Fresh embryo transfer was canceled if the patient had an unfavorable endometrium, (endometrial thickness of ≤ 6 mm or ≥ 16 mm, fluid in cavity or endometrial polyp), progesterone level ≥ 1.5 ng/mL on the triggering day or high risk of ovarian hyperstimulation syndrome (OHSS). Two good quality D3 embryos were frozen then. All surplus embryos were cultured for two or three more days, and good-quality blastocysts were vitrified.

Luteal phase support in fresh embryo transfer cycles was initiated from the day of OPU with 10 mg dydrogesterone twice daily and 90mg progesterone sustained-release vaginal gel (Crinone, Merck Serono, Germany). For frozen embryo transfer (FET), the endometrium was prepared through a natural or artificial cycle regimen based on the doctor’s decision. Once the patients were pregnant, the luteal phase support continued until 7 weeks of gestation for natural cycle FET, and 10–12 weeks of gestation for fresh and artificial cycle FET.

### Outcomes and measures

Positive βhCG was defined as plasma βhCG >10 IU/L 10–14 days after embryo transfer. Clinical pregnancy was confirmed by observing a gestational sac by ultrasonography 20 more days later. Ongoing pregnancy was defined as visible fetal heart activity on ultrasonography from 12 weeks of gestation onwards. Then the patients were followed up until live birth.

Ovarian ultrasound scans were performed using a 5.0–9.0 MHz multi-frequency transvaginal probe to evaluate the number and sizes of antral follicles. AFC was calculated as the number of all follicles measuring 3–8 mm in diameter at baseline. PFC was defined as the number of follicles measuring 16–22 mm in diameter in both ovaries on the trigger day. Average Gn dosage per follicle was calculated as the total administered FSH dosage/PFC. FORT was calculated as PFC/AFC. FOI was calculated as the total number of oocytes retrieved/ AFC. OSI was calculated as (the number of oocytes retrieved/ total Gn dose) × 1000. Follicular sensitivity index (FSI) was calculated as PFC × 100,000/ (AFC × total received FSH doses).

### Blood samples and hormone assays

Serum P, E_2_, LH and FSH levels were measured using a competitive chemiluminescence immunoassay using commercial kits obtained from Roche Diagnostics. Blood test was performed at a relatively fixed time, i.e., between 8 a.m. and half past 8 a.m., to minimize the possible influence of circadian rhythm changes on hormone levels. All measurements were performed according to the manufacturer’s instructions.

### Statistical analysis

Continuous data are expressed as means ± standard deviations. Between-group differences were tested by ANOVA or Kruskal Wallis rank test. Categorical data are presented as frequency and percentage; differences were assessed by the chi-squared test or Fisher’s exact test. The cumulative live birth rate (CLBR) in an entire ART cycle was assessed crudely and using multivariate logistic regression analysis. The decision to add each potential confounding factor to the model was based on previous scientific evidence and the results in the unadjusted analyses.

We also did sensitivity analyses for the baseline characteristics and demographic data, cycle parameters and indexes for ovarian response between patients who got cumulative live birth (CLB) and did not get CLB.

All analyses were done with the IBM SPSS Statistics for Windows, Version 22.0 (IBM Corp., Armonk, NY, USA). A *P *value < 0.05 was considered statistically significant.

## Results

Clinical data was retrieved from computerized clinical database and review of patients’ clinical records. Baseline characteristics and demographic data were compared among the three groups (Table 1). The three subgroups, formed on the basis of the average Gn dosage per follicle, significantly differed for age, basal FSH, and AFC. Age and basal FSH were progressively higher from Group A to Group C; while Group C had an AFC value significantly lower than the other groups (*P* < 0.01).

**Table 1:** Baseline patient characteristics and demographic data

N	Group A	Group B	Group C	*P*
	110	110	110	
Duration of infertility (years)	2.78 ± 1.99	2.82 ± 2.13	2.70 ± 1.75	0.89
Age (years)	30.83 ± 3.06	31.35 ± 3.63	32.26 ± 3.09	< 0.01
Basal FSH (IU/L)	6.59 ± 2.06	6.75 ± 1.93	7.41 ± 2.06	< 0.01
Basal LH (IU/L)	4.72 ± 2.05	4.44 ± 1.72	4.44 ± 1.78	0.44
Basal E2 (pg/ml)	46.04 ± 16.27	46.53 ± 14.24	43.89 ± 14.89	0.39
Basal P (ng/ml)	0.38 ± 0.20	0.38 ± 0.23	0.37 ± 0.23	0.92
AFC	18.97 ± 5.37	16.11 ± 3.88	12.23 ± 4.16	< 0.01
BMI (kg/m^2^)	22.26 ± 2.79	22.56 ± 3.19	22.30 ± 3.35	0.74
Diagnosis				0.25
Primary infertility	74 (67.27%)	82 (74.55%)	71 (64.55%)	
Secondary infertility	36 (32.73%)	28 (25.45%)	39 (35.45%)	
Indications				0.69
Tubal factor	70 (63.64%)	73 (66.36%)	70 (63.64%)	
Endometriosis	0 (0.00%)	1 (0.91%)	2 (1.82%)	
Unexplained infertility	23 (20.91%)	26 (23.64%)	25 (22.73%)	
Male factor	11 (10.00%)	5 (4.55%)	10 (9.09%)	
Others	6 (5.45%)	5 (4.55%)	3 (2.73%)	

*FSH* follicle stimulating hormone, *LH* luteinizing hormone, *E*_*2*_ estradiol, *AFC* antral follicle count, *BMI* body mass index, *Group A* below the 33rd percentile, *Group B* between 33rd and 67th percentiles, *Group C* above the 67th percentile

As shown in Table 2, many ovarian stimulation characteristics and laboratory parameters were significantly different among the three groups. With less total Gn administered and a shorter duration, patients in Group A exhausted the least Gn dosage per follicle. However, the FORT, FOI, and OSI were the highest for these patients, which meant the best ovarian response. The number of retrieved oocytes in Group A was significantly higher than in Group B and C. Moreover, Group B also had more oocytes retrieved than Group C. The number of good quality embryos on D3 also showed decreasing trend from Group A to Group C and prominently lower in Group C than in the other two groups (*P* < 0.01) (Table 2).

**Table 2:** Cycle parameters of patients grouped according to average Gn dosage per follicle

N	Group A	Group B	Group C	*P*
	110	110	110	
Initial Gn dose (IU)	199.89 ± 30.41	223.18 ± 33.30	243.64 ± 40.73	< 0.01
Total Gn dose (IU)	1607.44 ± 296.70	1952.96 ± 427.61	2443.64 ± 563.58	< 0.01
Gn duration (days)	9.24 ± 0.94	9.44 ± 1.11	9.97 ± 1.44	< 0.01
LH on day of trigger (IU/L)	2.42 ± 1.73	2.28 ± 1.45	2.74 ± 1.76	0.11
E_2_ on day of trigger (pg/ml)	4176.34 ± 1681.63	3207.76 ± 1424.61	2452.90 ± 1154.19	< 0.01
P on day of trigger (ng/ml)	0.81 ± 0.41	0.82 ± 0.41	0.75 ± 0.43	0.46
Endometrial thickness (mm)	10.43 ± 2.33	10.68 ± 2.08	10.63 ± 2.17	0.67
FORT	0.98 ± 0.36	0.77 ± 0.23	0.70 ± 0.26	< 0.01
FOI	1.03 ± 0.46	0.90 ± 0.35	0.93 ± 0.39	0.04
OSI	11.80 ± 4.31	7.34 ± 2.46	4.64 ± 2.09	< 0.01
No. of oocytes	18.52 ± 6.12	14.09 ± 5.04	10.79 ± 4.21	< 0.01
No. of fertilized	14.40 ± 5.86	10.68 ± 4.38	8.41 ± 3.92	< 0.01
No. of 2PN	11.25 ± 5.18	8.64 ± 4.36	6.46 ± 3.65	< 0.01
No. of good quality embryos	4.75 ± 3.57	4.21 ± 3.04	2.86 ± 2.09	< 0.01

*Gn* gonadotropin, *FORT* follicular output rate = No. of pre-ovulatory follicles/AFC, *FOI* follicle-to-oocyte index = No. of oocytes retrieved/AFC, *OSI* ovarian sensitivity index = number of retrieved oocytes/total Gn dose × 1000; *2PN* two pronuclei

Analyses of the cumulative pregnancy outcomes showed that the rates of positive βhCG, clinical pregnancy, ongoing pregnancy, and live birth were significantly different among the three Groups. The results showed that cumulative pregnancy outcomes were the poorest for patients in Group C (Table 3). Table 4 summarized the results of a multivariate regression analysis. The results showed that in addition to AFC, number of oocytes retrieved, number of good quality embryos, FOI, and OSI, average Gn dosage per follicle was the most valuable independent predictor of CLBR [adjusted odds ratio (OR): 0.96, 95% confidence interval (CI): 0.95–0.98, *P* < 0.01].

**Table 3:** Cumulative pregnancy outcomes among the three groups based on average Gn dosage per follicle

	Group A	Group B	Group C	*P*
Positive βhCG	84 (76.36%)	92 (83.64%)	66 (60.00%)	< 0.01
Clinical pregnancy	80 (72.73%)	89 (80.91%)	65 (59.09%)	< 0.01
Ongoing pregnancy	76 (69.09%)	83 (75.45%)	58 (52.73%)	< 0.01
Live birth	75 (68.18%)	83 (75.45%)	55 (50.00%)	< 0.01
Biochemical miscarriage	4 (4.76%)	3 (3.26%)	1 (1.52%)	0.54
First trimester pregnancy loss	4 (5.00%)	6 (6.74%)	7 (10.77%)	0.40
Second trimester pregnancy loss	1 (1.32%)	0 (0.00%)	3 (5.17%)	0.07

Data were presented as n (%). Statistical analysis was carried out using Chi-square test

**Table 4:** Univariate and multivariate regression analysis of factors related to the cumulative live birth

Parameters	Univariate OR (95%CI)	*P*	Multivariate OR^*^ (95% CI)	*P*
AFC	1.07 (1.02–1.12)	< 0.01	1.07 (1.02–1.12)	< 0.01
No. of oocytes	1.13 (1.07–1.18)	< 0.01	1.12 (1.07–1.18)	< 0.01
No. of good quality embryos	1.38 (1.24–1.54)	< 0.01	1.39 (1.24–1.55)	< 0.01
FORT	1.30 (0.62–2.75)	0.49	1.26 (0.59–2.67)	0.54
FOI	2.69 (1.34–5.38)	< 0.01	2.60 (1.30–5.23)	< 0.01
OSI	1.18 (1.10–1.27)	< 0.01	1.18 (1.10–1.27)	< 0.01
average Gn dose per follicle	0.96 (0.94–0.98)	< 0.01	0.96 (0.95- 0.98)	< 0.01

^*^ ORs are adjusted by age and BMI

Results of the sensitivity analyses were shown in Additional file 1: Table S1. Patients who achieved CLB had lower basal FSH and higher AFC value, which meant a better ovarian reserve. With less total Gn dose and shorter duration, these patients also showed a better ovarian response. FORT showed no significant difference between patients who achieved CLB and not. Whereas, FOI and OSI were significantly higher in patients who achieved CLB. The results were consistent with which of average Gn dosage per follicle proposed in this study (236.48 ± 147.61 vs. 177.73 ± 108.71,* P* < 0.01).

## Discussion

Accurate prediction of ovarian response is crucial for most optimal and individualized ovarian stimulation. Clinicians can also provide better advice to patients and predict the risk of adverse events after ovarian stimulation, such as prolonged cycle time, poor ovarian response and cancellation of cycle, or OHSS. Biological and biochemical markers such as AFC and AMH have been proven to predict both the poor and hyper ovarian response with fairly good accuracy [2]. However, previous studies demonstrated that these biomarkers represent a “static” snapshot of the individual ovarian reserve which do not properly reflect the “dynamic” nature of follicular growth in response to exogenous ovarian stimulation [4].

FORT was first introduced by Genro et al. in 2011 to evaluate ovarian response during stimulation [6]. Some studies suggested that FORT < 0.30 indicated low ovarian sensitivity [6, 7]. It was one of the classic indexes to evaluate ovarian response, and has been studied in different populations and different ovarian stimulation protocols. Hassan A et. al concluded that FORT is an independent variable affecting the clinical pregnancy rate in IVF/ICSI cycles. Higher FORT values had better oocyte yield and clinical pregnancy rates in women with unexplained infertility undergoing IVF/ICSI with potentially normal ovarian response [13]. For patients with polycystic ovary syndrome (PCOS) undergoing IVF-ET, FORT was also a powerful tool for measuring ovarian reactivity. A high FORT to obtain high-quality embryos and perform FET could achieve good pregnancy outcome [14]. For the care and management of hypo-responders in ART, FORT proved to be a relevant and crucial quantitative, and qualitative index. Impaired sensitivity to FSH revealed by FORT should be considered in the decision of treatment protocol, gonadotropin, and stimulation doses to be used for hypo-responders [15]. However, this index had some drawbacks that should not be ignored. FORT did not assess the actual number of oocytes retrieved, which was strongly associated with live birth rates [1]. Moreover, FORT implied that 3–8 mm follicles before stimulation responded coordinately to FSH. It was not always the case. However, the individual tracking of the development of each follicle in response to FSH was practically unrealistic.

In 2018, the FOI was proposed to address the ovarian sensitivity. FOI ≤ 0.50 indicated low ovarian sensitivity and FOI > 0.50 for normal ovarian sensitivity. Hypo-responsiveness and suboptimal/poor response were not synonymous. FOI might be used alone or combined with FORT to most optimally reflect the ovarian response to Gn. However, FOI could be influenced by the initial Gn dosage, genetic or environmental factors, asynchronous follicular development, and technical issues during triggering and OPU [4]. Technical aspects related to oocyte retrieval and triggering for final oocyte maturation can influence FOI results. And it should also be taken into account in patients with low FOI.

Another marker of the ovarian potential to produce oocytes in response to hormonal stimulation was OSI, which was calculated by dividing the total administered FSH dose and the number of retrieved oocytes. It was first developed by Biasoni et al. and the definition was modified later [8, 16, 17]. The OSI was an interesting tool to assess the ovarian sensitivity to exogenous Gn and could be used to adjust the stimulation regimen in subsequent IVF cycles. A retrospective comparative cohort study including a total of 2150 women who underwent the first IVF cycle using long-agonist protocol validated the use of OSI as a highly reliable index of ovarian responsiveness to recombinant FSH and could be useful to estimate the FSH dose [18]. Another retrospective cohort study, with patients ≥ 39 years who performed their first ART cycle with an antagonist protocol, suggested that OSI was the best index to predict cumulative implantation rate and CLBR. Both OSI and FOI predicted embryo culture with success, but OSI was more accurate [19]. However, The OSI does not consider the Gn type (e.g., whether LH or LH analog was added) or the AFC. Besides, similar with FOI, technical aspects related to oocyte retrieval and triggering for final oocyte maturation could influence the number of oocytes retrieved and confuse its interpretation.

FSI as a new tool for objective assessment of follicular responsiveness to exogenous gonadotropins was proposed in 2017. Hassan AMA et al. demonstrated that FSI could predict the clinical pregnancy rate in women with unexplained infertility or tubal factor undergoing IVF/ICSI using GnRH agonist protocol. Higher FSI values had significantly higher oocyte yield and fertilization and clinical pregnancy rates [20]. However, the use of FSI may have some limitations in practice. For example, in women with PCOS, the use of FSI will be limited by the high AFC. Till now, no further large-scale studies have been performed to validate the utilization the FSI as a predictor of ovarian response to exogenous gonadotropins.

Taken together, there is no single perfect indicator for ovarian response, which can be affected by many factors. Polymorphism of FSHR, LHR, exons of the LH gene, and sequence variants in the genes that participate in estrogen synthesis such as CYP19A1, were all related to the ovarian response to exogenous Gn, even CLB [21–24]. Besides, other factor, such as oxidative stress in the follicular environment, was also associated with ovarian response [25]. In clinical practice, more indexes are needed to be explored or combined with other indexes, to better predict ovarian response and further reproductive outcomes.

The new marker in this study, named average Gn dosage per follicle, links the number of pre-ovulatory follicles on the trigger day to the degree of hormonal stimulation, expressing how many units of exogenous Gn are needed to obtain each follicle. It can allow doctors to avoid the influence of OPU techniques, inappropriate trigger timing and other factors. Thus, it may be used singularly or combined with other indexes to better assess ovarian response. In this retrospective study, patients with the lowest value of this index (Group A) had the lowest age, and the "static" indexes of ovarian reserve such as basic FSH and AFC were the best. Meanwhile, with the least total Gn dose and shortest Gn duration, the most oocytes and good quality embryos were retrieved. Other indexes of ovarian response, FORT, FOI, and OSI were also the best. As to the cumulative pregnancy outcomes of an entire ART cycle, patient with low (Group A) and medium (Group B) Gn dosage per follicle had more chances of getting pregnant or live birth, compared with patients with high value of this index (Group C). In a multivariate regression model, the average Gn per follicle was also shown to represent an independent predictor of CLB.

In this study, we also carried on another comparison and analysis. Patients were divided into two groups based on whether CLB was achieved in an entire ART cycle. Then baseline characteristics and demographic data, cycle parameters and indexes for ovarian response were compared. The results showed that patients who achieved CLB had better "static" markers of ovarian reserve, such as basal FSH and AFC, and higher "dynamic" markers of ovarian response, such as FOI and OSI, and lower average Gn dosage per follicle. Although FORT was not statistically different between the two groups, it was also observed that the FORT was slightly higher in patients with CLB, which could not be ruled out as being related to the small sample size.

This study also has some limitations arising from the retrospective nature. Firstly, patients in the study were stimulated with GnRH antagonist protocol, whether the conclusions could be extrapolated to other ovarian stimulation protocols needs further study. Secondly, we have studied herein a relatively small cohort of patients, although quite homogeneous. Therefore, these observations need to be interpreted with caution. Further studies with broader inclusion criteria and more personalized protocols are needed to validate these results. Thirdly, this index, average Gn dosage per follicle, assumed that only 16–22 mm follicles on trigger day effectively responded to FSH, while it is conceivable that smaller follicles also presented certain degree of FSH responsiveness. And those 14–15 mm follicles on trigger day may have also reached their FSH-driven maturation. Further studies are needed to verify if this index is still instrumental when those intermediate-sized follicles are included into calculation except for the 16–22 mm follicles on trigger day.

Taken together, larger scale multi-center prospective studies are required to further verify the utility of this index. Future studies, evaluating alternative ways of calculating the index, including patients of different ovarian reserve and treated with different protocols, and incorporating this index into more complex prediction models of IVF outcomes, will undoubtedly contribute to broaden its clinical applications.

## Conclusion

In conclusion, this study suggested that the average Gn dosage per follicle, consistent with other classical indicators, reflected ovarian response quite precisely, and might be used to predict the probability of CLB.

## Supplementary Information

**Additional file1.** Sensitivity analyses between patients who got cumulative live birth (CLB) and did not get CLB.

## Data Availability

All data is available in this paper.

## Abbreviations

ART: Assisted reproductive technology
IVF: In vitro fertilization
Gn: Gonadotrophin
LBR: Live birth rate
FSH: Follicle-stimulating hormone
E_2_: Estradiol
AMH: Anti-Mullerian hormone
AFC: Antral follicle count
FORT: Follicle output rate
FOI: Follicle-to-oocyte index
OSI: Ovarian sensitivity index
FSI: Follicular sensitivity index
PFC: Pre-ovulatory follicle count
BMI: Body mass index
GnRH: Gonadotropin releasing hormone
OPU: Ovum pick-up
OHSS: Ovarian hyperstimulation syndrome
FET: Frozen embryo transfer
CLBR: Cumulative live birth rate
CLB: Cumulative live birth
OR: Odds ratio
CI: Confidence interval

## References

[1] Sunkara SK, Rittenberg V, Raine-Fenning N, Bhattacharya S, Zamora J, Coomarasamy A (2011). Association between the number of eggs and live birth in IVF treatment: an analysis of 400 135 treatment cycles. Hum Reprod.

[2] La Marca A, Sunkara SK (2014). Individualization of controlled ovarian stimulation in IVF using ovarian reserve markers: from theory to practice. Hum Reprod update.

[3] Pezzuto A, Ferrari B, Coppola F, Nardelli GB (2010). LH supplementation in down-regulated women undergoing assisted reproduction with baseline low serum LH levels. Gynecol Endocrinol.

[4] Alviggi C, Conforti A, Esteves SC, Vallone R, Venturella R, Staiano S (2018). Understanding ovarian hypo-response to exogenous gonadotropin in ovarian stimulation and its new proposed marker-the follicle-to-oocyte (FOI) index. Front Endocrinol (Lausanne).

[5] Conforti A, Esteves SC, Cimadomo D, Vaiarelli A, Di Rella F, Ubaldi FM (2019). Management of women with an unexpected low ovarian response to gonadotropin. Front Endocrinol (Lausanne).

[6] Genro VK, Grynberg M, Scheffer JB, Roux I, Frydman R, Fanchin R (2011). Serum anti-Müllerian hormone levels are negatively related to Follicular Output RaTe (FORT) in normo-cycling women undergoing controlled ovarian hyperstimulation. Hum Reprod.

[7] Gallot V, Berwanger da Silva AL, Genro V, Grynberg M, Frydman N, Fanchin R (2012). Antral follicle responsiveness to follicle-stimulating hormone administration assessed by the Follicular Output RaTe (FORT) may predict in vitro fertilization-embryo transfer outcome. Hum Reprod.

[8] Biasoni V, Patriarca A, Dalmasso P, Bertagna A, Manieri C, Benedetto C (2011). Ovarian sensitivity index is strongly related to circulating AMH and may be used to predict ovarian response to exogenous gonadotropins in IVF. Reprod Biol Endocrinol.

[9] De Placido G, Alviggi C, Perino A, Strina I, Lisi F, Fasolino A (2005). Recombinant human LH supplementation versus recombinant human FSH (rFSH) step-up protocol during controlled ovarian stimulation in normogonadotrophic women with initial inadequate ovarian response to rFSH. A multicentre, prospective, randomized controlled trial. Hum Reprod.

[10] Ferraretti AP, Gianaroli L, Magli MC, D'Angelo A, Farfalli V, Montanaro N (2004). Exogenous luteinizing hormone in controlled ovarian hyperstimulation for assisted reproduction techniques. Fertil Steril.

[11] Ruvolo G, Bosco L, Pane A, Morici G, Cittadini E, Roccheri MC (2007). Lower apoptosis rate in human cumulus cells after administration of recombinant luteinizing hormone to women undergoing ovarian stimulation for in vitro fertilization procedures. Fertil Steril.

[12] Carosso AR, van Eekelen R, Revelli A, Canosa S, Mercaldo N, Benedetto C (2022). Women in advanced reproductive age: are the follicular output rate, the follicle-oocyte index and the ovarian sensitivity index predictors of live birth in an IVF cycle?. J Clin Med.

[13] Hassan A, Kotb M, AwadAllah A, Wahba A, Shehata N (2017). Follicular output rate can predict clinical pregnancy in women with unexplained infertility undergoing IVF/ICSI: a prospective cohort study. Reprod Biomed Online.

[14] Yang H, Lin J, Jin C, Meng L, Wu S, Chen Y (2020). The predictive value of the follicular output rate on pregnancy outcome of patients with polycystic ovary syndrome undergoing in vitro fertilization and embryo transfer. Med Sci Monit.

[15] Grynberg M, Labrosse J (2019). Understanding follicular output rate (FORT) and its implications for POSEIDON criteria. Front Endocrinol (Lausanne).

[16] Weghofer A, Barad DH, Darmon SK, Kushnir VA, Albertini DF, Gleicher N (2020). The ovarian sensitivity index is predictive of live birth chances after IVF in infertile patients. Hum Reprod Open..

[17] Huber M, Hadziosmanovic N, Berglund L, Holte J (2013). Using the ovarian sensitivity index to define poor, normal, and high response after controlled ovarian hyperstimulation in the long gonadotropin-releasing hormone-agonist protocol: suggestions for a new principle to solve an old problem. Fertil Steril.

[18] Yadav V, Malhotra N, Mahey R, Singh N, Kriplani A (2019). Ovarian Sensitivity Index (OSI): Validating the use of a marker for ovarian responsiveness in IVF. J Reprod Infertil.

[19] Cesarano S, Pirtea P, Benammar A, De Ziegler D, Poulain M, Revelli A (2022). Are there ovarian responsive indexes that predict cumulative live birth rates in women over 39 years?. J Clin Med.

[20] Hassan AMA, Kotb MMM, AwadAllah AMA, Shehata NAA, Wahba A (2017). Follicular sensitivity index (FSI): a novel tool to predict clinical pregnancy rate in IVF/ICSI cycles. J Assist Reprod Genet.

[21] Lazaros LA, Hatzi EG, Pamporaki CE, Sakaloglou PI, Xita NV, Markoula SI (2012). The ovarian response to standard gonadotrophin stimulation depends on FSHR, SHBG and CYP19 gene synergism. J Assist Reprod Genet.

[22] Song D, Huang XL, Hong L, Yu JM, Zhang ZF, Zhang HQ (2019). Sequence variants in FSHR and CYP19A1 genes and the ovarian response to controlled ovarian stimulation. Fertil Steril.

[23] Davar R, Tabibnejad N, Kalantar SM, Sheikhha MH (2014). The luteinizing hormone beta-subunit exon 3 (Gly102Ser) gene mutation and ovarian responses to controlled ovarian hyperstimulation. Iran J Reprod Med.

[24] Lindgren I, Nenonen H, Henic E, Bungum L, Prahl A, Bungum M (2019). Gonadotropin receptor variants are linked to cumulative live birth rate after in vitro fertilization. J Assist Reprod Genet.

[25] Uppangala S, Fernandes G, Salian SR, Kumar P, Talevi R, Kalthur G (2020). Reduced ovarian response to controlled ovarian stimulation is associated with increased oxidative stress in the follicular environment. Reprod Biol.

