# Supplementary material for: The average gonadotrophin dosage per follicle is predictive of ovarian response and cumulative live birth chances after in vitro fertilization: a retrospective cohort study
Source: BMC Womens Health. 2023 Feb 4;23:45. doi: 10.1186/s12905-023-02195-5 (PMC9898889; doi:10.1186/s12905-023-02195-5)
Supplement: Supplementary file 1 — Additional file1. Sensitivity analyses between patients who got cumulative live birth (CLB) and did not get CLB. [file 12905_2023_2195_MOESM1_ESM.docx]

**Supplementary Table 1 Baseline characteristics and outcomes of ovarian stimulation between patients who got CLB and did not get CLB in an entire ART cycle**

|  | without CLB | CLB | *P* |
| --- | --- | --- | --- |
| N | 117 | 213 |  |
| Age (years) | 31.82 ± 3.24 | 31.29 ± 3.35 | 0.16 |
| Indications |  |  | 0.53 |
| Tubal factor | 72 (61.54%) | 141 (66.20%) |  |
| Endometriosis | 2 (1.71%) | 1 (0.47%) |  |
| Unexplained infertility | 27 (23.08%) | 47 (22.07%) |  |
| Male factor | 12 (10.26%) | 14 (6.57%) |  |
| others | 4 (3.42%) | 10 (4.69%) |  |
| Basal FSH (IU/L) | 7.33 ± 2.16 | 6.69 ± 1.94 | <0.01 |
| Basal LH (IU/L) | 4.58 ± 1.68 | 4.51 ± 1.95 | 0.75 |
| Basal E_2_ (pg/ml) | 45.09 ± 14.87 | 45.70 ± 15.34 | 0.72 |
| Basal P (ng/ml) | 0.38 ± 0.19 | 0.38 ± 0.23 | 0.91 |
| AFC | 14.67 ± 5.34 | 16.38 ± 5.17 | <0.01 |
| BMI (kg/m^2^) | 22.52 ± 3.24 | 22.30 ± 3.04 | 0.53 |
| Total Gn dose (IU) | 2108.38 ± 600.87 | 1942.55 ± 527.69 | 0.01 |
| Gn duration (days) | 9.79 ± 1.35 | 9.42 ± 1.12 | <0.01 |
| FORT | 0.80 ± 0.30 | 0.82 ± 0.31 | 0.49 |
| FOI | 0.87 ± 0.42 | 1.00 ± 0.38 | <0.01 |
| OSI | 6.35 ± 3.63 | 8.79 ± 4.38 | <0.01 |
| Average Gn dose per follicle (IU) | 236.48 ± 147.61 | 177.73 ± 108.71 | <0.01 |
| No. of oocytes | 12.12 ± 5.55 | 15.76 ± 5.95 | <0.01 |
| No. of fertilized | 9.01 ± 5.00 | 12.35 ± 5.22 | <0.01 |
| No of 2PN | 6.72 ± 4.40 | 9.92 ± 4.71 | <0.01 |
| No. of good quality embryos on D3 | 2.55 ± 2.17 | 4.70 ± 3.21 | <0.01 |
